# Molecular and serological epidemiology of *Leptospira* infection in cats in Okinawa Island, Japan

**DOI:** 10.1038/s41598-021-89872-3

**Published:** 2021-05-14

**Authors:** Tetsuya Kakita, Yumani Kuba, Hisako Kyan, Sho Okano, Masatomo Morita, Nobuo Koizumi

**Affiliations:** 1 Department of Biological Sciences, Okinawa Prefectural Institute of Health and Environment, 17-1 Kanekadan, Uruma-shi, Okinawa, 904-2241 Japan; 2 Department of Bacteriology I, National Institute of Infectious Disease, 1-23-1 Toyama, Shinjuku-ku, Tokyo, 162-8640 Japan; 3 Present Address: Regional Health Division, Department of Public Health and Medical Care, Okinawa Prefectural Government, 1-2-2 Izumizaki, Naha-shi, Okinawa, 900-8570 Japan

**Keywords:** Bacterial infection, Infectious-disease epidemiology

## Abstract

Leptospirosis is a zoonosis caused by pathogenic *Leptospira* spp. Cats have been reported to be infected with *Leptospira* spp. and shed the bacteria in the urine. However, the importance of cats as an infection source for humans remains unclear. In this study, *Leptospira* infection in cats in Okinawa Prefecture, Japan, where leptospirosis is endemic, was investigated by leptospiral antibody and DNA detection using microscopic agglutination test and nested PCR, respectively. Moreover, multilocus sequence typing (MLST) and whole genome sequencing (WGS) were conducted on the *Leptospira borgpetersenii* serogroup Javanica isolated from cats, black rats, a mongoose, and humans. Anti-*Leptospira* antibodies were detected in 16.6% (40/241) of the cats tested, and the predominant reactive serogroup was Javanica. The leptospiral *flaB* gene was detected in 7.1% (3/42) of cat urine samples, and their sequences were identical and identified as *L. borgpetersenii*. MLST and WGS revealed the genetic relatedness of *L. borgpetersenii* serogroup Javanica isolates. This study indicated that most seropositive cats had antibodies against the serogroup Javanica and that cats excreted *L. borgpetersenii* in the urine after infection. Further, genetic relatedness between cat and human isolates suggests that cats may be a maintenance host for *L. borgpetersenii* serogroup Javanica and a source for human infection.

## Introduction

Leptospirosis is a zoonotic disease caused by infection with pathogenic spirochetes of the genus *Leptospira*, composed of 64 species divided into 24 serogroups and more than 300 serovars^1–4^. *Leptospira* spp. colonize the proximal renal tubules of maintenance hosts, including wild animals such as rats and boars, livestock such as cattle and pigs, and companion animals such as dogs, and are shed in their urine^2,5,6^. Humans are infected percutaneously or permucosally with *Leptospira* spp. by direct contact with the urine of maintenance hosts or by indirect contact with soil or water contaminated with infected urine^3,6,7^.

Cats have not been considered an important source of *Leptospira* infection for humans. Besides antibody detection, however, several recent studies from various regions reported that *Leptospira* spp. were isolated or leptospiral DNAs were detected from cat urine or kidney samples^8–21^; carriage rate was related to some factors such as rearing style (cat external behavior), climate, and living environment (urban or rural)^15,20^. Moreover, leptospiral DNA was continuously detected in the urine of naturally infected cats for 8 months^20^. Although symptomatic cases with polyuria, polydipsia, hematuria, ascites, and diarrhea have been reported, the clinical presentation of leptospirosis in cats is rare, usually mild, or subclinical, and symptoms in feline leptospirosis remain undefined^9,13,14,17,20,22^. These studies suggest that cats can carry and shed pathogenic *Leptospira* in urine for a long period after infection. Asymptomatic or uncertain symptoms of infected cats make the diagnosis and appropriate treatment difficult and prevent infected cats from becoming a chronic carrier, resulting in a potential infection source for humans.

Cats are the most common companion animal in Japan. According to a survey conducted by the Japan Pet Food Association, the number of cats has been increasing year by year, and the number of cats raised in Japan is estimated to be 9,778,000 in 2019, higher than that of dogs^23^. Canine leptospirosis and its causative *Leptospira* spp. have been recently reported^24,25^, but the current status of *Leptospira* infection in cats in Japan remains limited. In the southern Kyushu District, the prevalence of anti-*Leptospira* antibodies in domestic cats was reported to be 7.7%^26^. In Okinawa Prefecture, the southernmost part of Japan, where leptospirosis is endemic, it was reported 30 years ago that the seroprevalence and isolation rate of *Leptospira* spp. among cats ranged from 4.8 to 9.1% and 1.0% to 3.1%, respectively^27,28^. Although *Leptospira* spp. were isolated, molecular characterization of the isolates was not performed in these studies.

Understanding the *Leptospira* genotype-host association in maintenance hosts is important for elucidating and controlling the source for human infection. Currently, molecular typing methods, such as multilocus sequence typing (MLST) and multilocus variable-number tandem repeat analysis (MLVA), are the main methods for characterizing *Leptospira* isolates^1^. As a genotyping method having higher resolution, whole genome sequencing (WGS) has been rapidly developed in recent years. These methods have enabled the understanding of host specificity of certain *Leptospira* genotypes as well as the geographic structuring of genetic diversity and host switching event in *Leptospira* spp^29–32^.

In this study, to clarify *Leptospira* infection and carriage in cats in Okinawa Island (Okinawa Main Island), anti-leptospiral antibodies in cats were investigated by the microscopic agglutination test (MAT) using 13 reference strains. Leptospiral DNA was detected from cat urine samples by nested PCR. Moreover, MLST and WGS were performed to gain further insights into the genetic relatedness of *Leptospira* isolates from cats, mongooses, rats, and humans.

## Results

Antibodies against *Leptospira* spp. were detected in 40 of 241 cats (16.6%) included in the analysis. Thirty-seven cats and one cat had antibodies against serogroups Javanica and Hebdomadis, respectively, and two samples were positive for multiple serogroups (Table 1). The reciprocal antibody titers for serogroup Javanica ranged from 160 to 2560 (Table 1). The antibody-positive samples in the northern, central, and southern regions of Okinawa Island were 15.0% (26/173), 22.0% (9/41), and 18.5% (5/27), respectively. Of the 40 anti-leptospiral antibody-positive cats, 23 were male (19.3%) and 17 were female (13.9%). There were no significant differences between seropositivity and capture area nor between seropositivity and sex. In contrast, the seropositivity varied among the age groups based on weight: 3% (1/33) in kittens, 12.1% (7/58) in juveniles, and 21.3% (32/150) in adults. There was a statistically significant difference in the seropositivity among the age groups (*p* = 0.021), and a trend toward an increase in seropositivity with age was observed (*p* = 0.006).

**Table 1:** Frequency of anti-*Leptospira* antibodies in cats.

Reciprocal antibody titer	160	320	640	1280	2560	No. of positives
**Serogroup**						
Javanica	13	7	7	6	4	37
Hebdomadis	0	0	1	0	0	1
Multiple	(2^†,‡^)		(1^†^)	(1^‡^)		2

^†^The cat FS13001 exhibited the reciprocal MAT titer 160 and 640 for serogroups Ballum and Javanica, respectively.

^‡^The cat FS18013 exhibited the reciprocal MAT titer 160 and 1280 for serogroups Autumnalis and Hebdomadis, respectively.

MAT, microscopic agglutination test.

**Figure 1:**
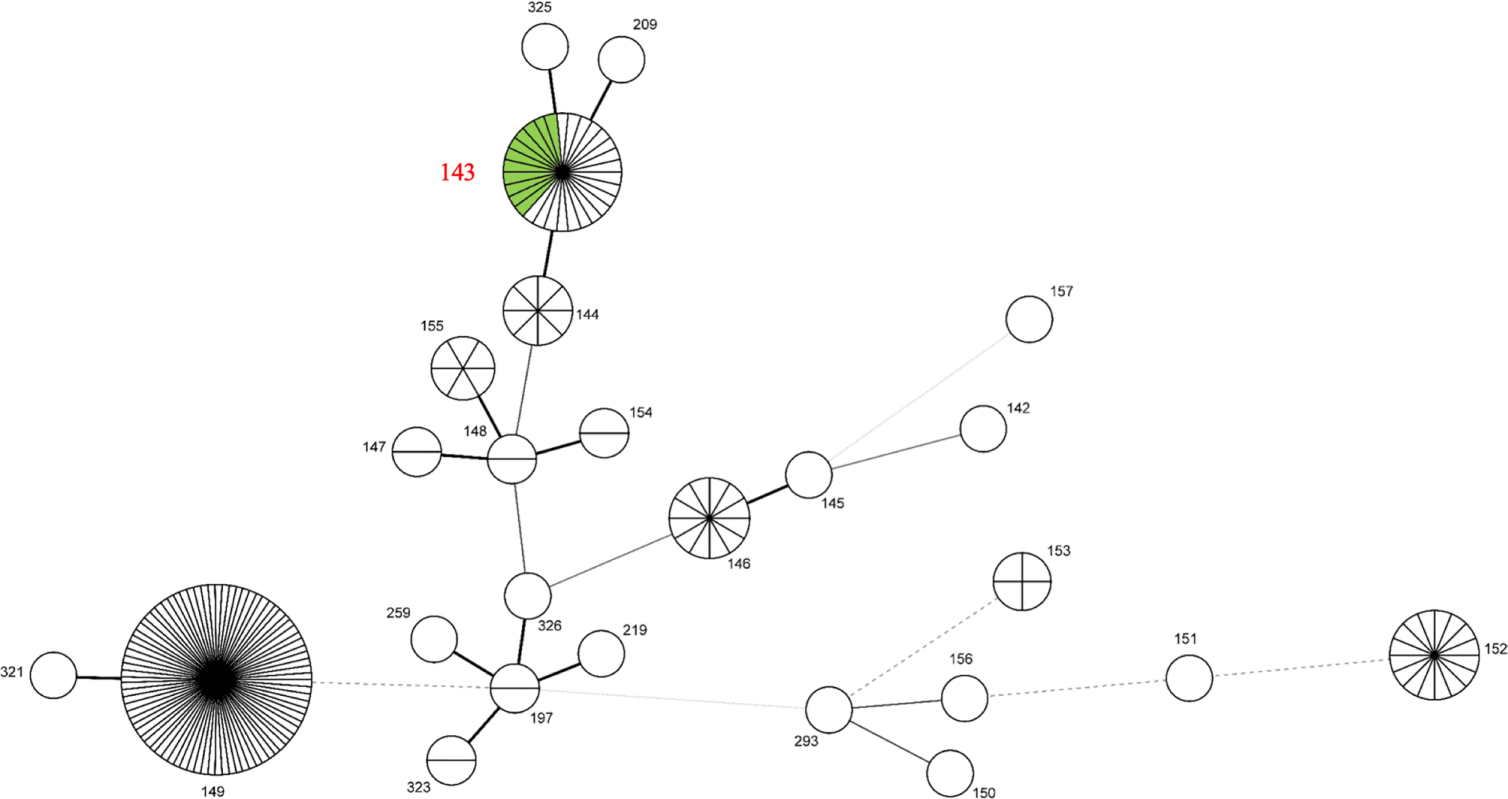
MST of *L. borgpetersenii* detected from cat urine samples and *L. borgpetersenii* serogroup Javanica strains in this study and 182 *L. borgpetersenii* strains based on the allelic profiles of the MLST seven housekeeping genes. Each circle represents an individual ST, and the numbers represent ST numbers. Circle sizes correspond to the numbers of strains in each ST. The thickness and the dotting of lines indicate the distance between the circles: a thicker line indicates a closer distance than a thin line, and a thin line denotes a closer distance than a dotted line. The green-colored circle and the ST number in red represent *L. borgpetersenii* serogroup Javanica strains and DNAs determined in this study.

Leptospiral *flaB* was detected in 3 of 42 urine samples (7.1%, see Supplementary Fig. S1 online) included in the analysis. All three PCR-positive cats were also positive for antibody against serogroup Javanica with the titers of 320 (2 animals) and 640 (1 animal). All three *flaB* sequences were identical and identified as *L. borgpetersenii* (DDBJ accession numbers LC596932–LC596934). Amplification of all seven housekeeping genes for MLST was succeeded in two of the three *flaB*-positive urine samples (FU18017 and FU18027) and nine strains of *L. borgpetersenii* serogroup Javanica (Table 2). Their sequence types (STs) were all assigned as ST143 (Fig. 1). There were no gross abnormalities in the autopsy findings in the three cats.

**Table 2:** *L. borgpetersenii* serogroup Javanica strains used for MLST and/or WGS.

Strain	Animal from which *Leptospira* strain was isolated	Year of isolation	Analytical method
FK-118	Cat	1989	MLST/WGS
F-208	Cat	1990	MLST/WGS
OHJ2008-88U	Small Indian mongoose	2008	MLST/WGS
OR2010-56	Black rat	2010	MLST
OR2010-61	Black rat	2010	MLST
OR2011-1	Black rat	2011	MLST
OP104	Human	2000	MLST/WGS
058031	Human	2005	MLST/WGS
078065	Human	2007	MLST/WGS

MLST, multilocus sequence typing; WGS, whole genome sequencing.

**Figure 2:**
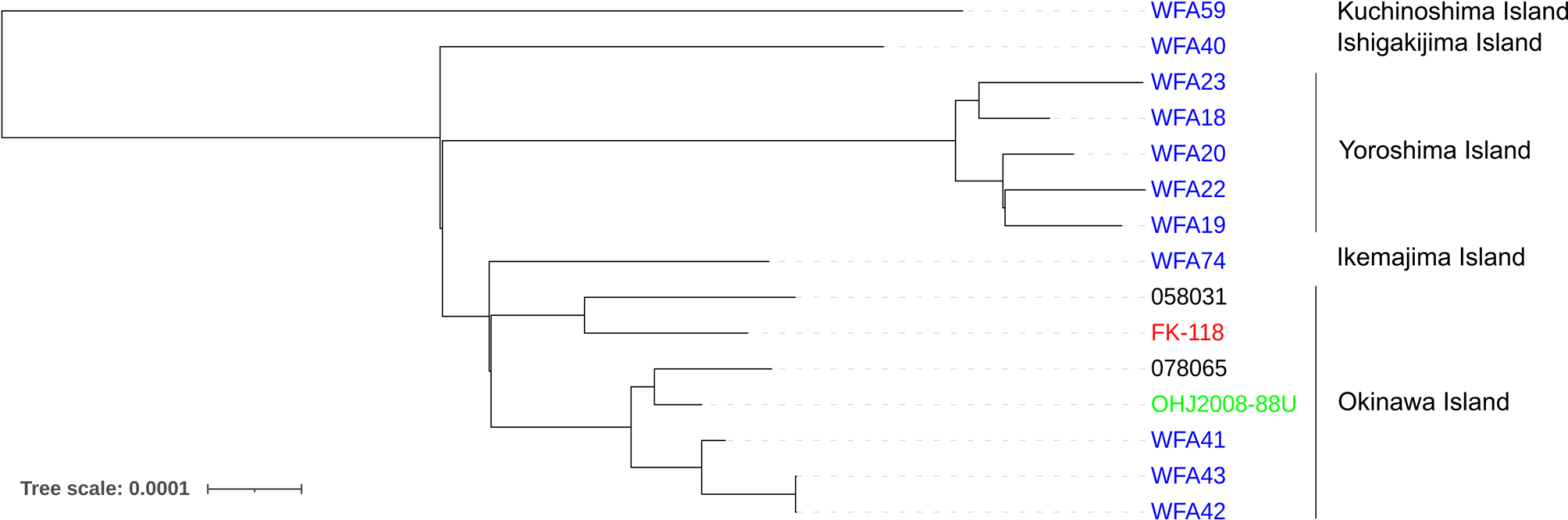
Core genome SNP-based maximum likelihood tree of *L. borgpetersenii* serogroup Javanica strains isolated in Japan. The strain Piyasena, belonging to serogroup Javanica serovar Ceylonica, was used as the reference, omitted from the tree. WGS of strains 058031, 078065, FK-118, and OHJ2008-88U was performed in this study, and strains named WFA have been analyzed in a previous study^30^. Black, human isolate; blue, black rat isolate; green, mongoose isolate; red, cat isolate.

Of the six strains subjected to WGS, enough data for comparison were obtained from four strains, OHJ2008-88U, FK-118, 058031, and 078065. Strains isolated from a cat, a mongoose, and humans were clustered with *L. borgpetersenii* serogroup Javanica strains isolated from black rats in Okinawa Island sequenced in a previous study^30^ (Fig. 2).

## Discussion

Identification of prevalent serovar(s) and their maintenance host(s) and seroprevalence in the host(s) is important for understanding the epidemiology of leptospirosis in a particular geographic region^33^. In the Okinawa Island, which has a subtropical climate, *Leptospira* spp. have been isolated or detected from various animals such as rat, mouse, shrew, mongoose, wild boar, and dog^24,34–36^. The animals excrete the bacteria into the environment where *Leptospira* spp. can survive and remain infective for several weeks^7^. Humans can get infected with *Leptospira* spp. through contact with the contaminated environment during recreational activities in rivers and agricultural activities^37^. Therefore, the approach of “One Health” recognizing the interconnection between humans, animals, and their shared environment is important to elucidate the epidemiology of leptospirosis in this island. In this study, we demonstrated that free-roaming stray cats in Okinawa Island were predominantly infected with *L. borgpetersenii* serogroup Javanica and shed the bacterium in urine, suggesting a potential role of cats in transmitting leptospirosis to humans.

The prevalence of anti-*Leptospira* antibodies and the detection of leptospiral DNA among cats worldwide range from 4 to 33% and 0% to 67.8%, respectively^9–16,18–21^. In this study, the seropositivity was 16.6%, and more than 90% of the cat serum samples reacted with serogroup Javanica (Table 1). Pathogenic *Leptospira* DNA was detected in three cat urine samples (7.1%, 3/42). The seropositivity in this study was slightly higher than that in previous studies done in Okinawa Prefecture (mainly in the Okinawa Island) 30 years ago (4.8% and 9.1%), but reactive serogroups were much diverse in the previous studies^27,28^. Previous studies reported that *Leptospira* spp. were isolated from 1.0% and 3.1% of the cats tested, lower than the DNA detection in this study, but success in isolation is generally lower than that in DNA detection. The positive rate of anti-leptospiral antibodies and leptospiral DNA detection in cats could be affected by various factors, such as age, rearing styles, season, geographical region, presence of maintenance hosts, panel of serovars, and/or cutoff value used for MAT, sensitivity, and specificity of the primer (and probe) set used for PCR^9,15,16,38^, which may be true for this study, and primer sets for other genes may be able to detect leptospiral DNA in more samples. This study employed reciprocal MAT titer 160 as the cutoff value because MAT titer 100 is generally accepted to indicate a previous infection^3,5^. Under this definition, there was no significant association between seropositivity and capture area in Okinawa Island. A previous report indicated that the prevalence of anti-leptospiral antibodies in cats was higher in rural areas than in urban areas^9^. The southern part of Okinawa Island is an urban area, and it changes to rural areas as it moves northward. In addition, human leptospirosis is often reported in the northern area due to recreational activities in rivers^37^. These facts suggest no regional difference in the risk of leptospiral infection in cats in Okinawa Island. The previous study mentioned above included only domestic cats^9^, whereas this study included free-roaming, stray cats. Stray cats have more risk of contact with maintenance hosts than pet cats, even in urban areas, resulting in discrepant results between the two studies. In addition, as more than 90% of seropositive cats had antibodies against serogroup Javanica, the transmission of *L. borgpetersenii* serogroup Javanica may easily occur between cats. A significant association between seropositivity and cat age groups based on weight was shown in this study (*p* = 0.021), and seropositivity tended to increase with age (*p* = 0.006). This is consistent with the previous report describing that older cats had higher seroprevalence due to an increased opportunity for exposure to the source of infection^16^.

Each serovar tends to be maintained in specific animal species: host-maintained infections of global importance are Icterohaemorrhagiae in the brown rat, Hardjo in cattle and sheep, Canicola in dogs, and Bratislava in pigs^5^. Infections with several serovars/serogroups have been identified in cats, such as Australis, Autumnalis, Ballum, Bataviae, Bratislava, Canicola, Copenhageni, Cynopteri, Grippotyphosa, Hardjo, Icterohaemorrhagiae, Javanica, Panama, Pomona, Pyrogenes, Rachmati, or Shermani^9,12,14–16,18–20^. A serological survey revealed that cats were predominantly infected with serogroup Javanica in Okinawa Island (Table 1). Serogroup Javanica has also been isolated from black rats and mongooses in the northern part of Okinawa Island^34^. MLST revealed that *L. borgpetersenii* detected in urine samples and isolated from cats, black rats, a mongoose, and humans all belonged to ST143. Although ST143 has been isolated from mongooses, *L. interrogans* serogroup Hebdomadis is the predominant strain isolated from mongooses^34^. Since cats are carnivorous and avoid water, they are more likely to be infected by rat predation than by waterborne infections^39^. These facts suggest an infection cycle between cats and rats in Okinawa Island. Monitoring acute leptospirosis in dogs as sentinels is suggested to aid in estimating the risk to humans in specific areas^40^. Cats may also act as sentinels, but their clinical manifestations seem to be less apparent than those of dogs^9,13,14,22^ and their implications may need further verification.

Conversely, all urine PCR-positive cats showed high antibody titer against serogroup Javanica, indicating that they shed leptospires in urine for some period after infection. Therefore, infected stray cats contaminate the environment and can be a source for infection to humans. In this study, more than 90% of seropositive cats had antibodies against serogroup Javanica (Table 1), although a variety of *Leptospira* serogroups/serovars exist on the island^24,34–37^. In cases of leptospirosis in humans on this island during 2007–2016, Hebdomadis was the most frequently detected serogroup (40.1%, 57/142), whereas Javanica was rare (2.8%, 4/142)^37^. Urine PCR-positive DNA samples showed the presence of *L. borgpetersenii* ST143, which was the same ST with cat isolates on this island; furthermore, the serogroup of these isolates was Javanica (Table 2). It has been reported that naturally infected cats subclinically shed leptospires (leptospiral DNAs) for 8 months after infection^20^. These results suggest that this genotype of *L. borgpetersenii* serogroup Javanica can be easily transmitted among cats and that cats may act as their maintenance host. Antibodies against serogroup Javanica were also detected from cats in Taiwan^12^. Although no genetic information on serogroup Javanica strain from cats was obtained, the same *L. borgpetersenii* serogroup Javanica genotype has been isolated from rats in Taiwan and in other Asian countries, such as China, Indonesia, Laos, Thailand, and Sri Lanka^30^ (PubMLST; https://pubmlst.org/organisms/leptospira-spp). These facts suggest that cats may carry *L. borgpetersenii* serogroup Javanica in other Asian countries.

In addition to MLST, WGS revealed that *L. borgpetersenii* serogroup Javanica isolated from a cat, a mongoose, black rats, and humans in Okinawa Island belonged to the same cluster, supporting their genetic relatedness and the geographic structuring of genetic diversity of *Leptospira* species as with the previous studies^30,31^ (Fig. 2). The previous report indicated that this genotype of *L. borgpetersenii* serogroup Javanica could infect various rodent species, suggesting that they are a generalist pathogen^30^. Moreover, this study supports this *L. borgpetersenii* serogroup Javanica as a generalist, as they can colonize the kidney tissues of cats and mongooses. In this study, only one cat and mongoose succeeded in WGS. WGS of more cat and mongoose isolates could identify animal species-specific characteristics, which may gain new insights into the mechanism of renal colonization and evolution of *Leptospira* spp. in different animals. It could also identify the precise animal source for human infection.

In conclusion, this study reports that cats are commonly infected with and excrete *L. borgpetersenii* serogroup Javanica that are genetically closely related to those isolated from black rats, mongooses, and humans in Okinawa Island, Japan. Although genetic relatedness suggests that black rats and mongooses are the source of infection for cats, a high proportion of serogroup Javanica infection and urinary excretion of *L. borgpetersenii* after infection also suggest that cats may be a maintenance host of *L. borgpetersenii* serogroup Javanica and the source for human infection.

## Methods

### Sample collection

There were 241 serum samples and 42 urine samples collected in Okinawa Island, the main island of Okinawa Prefecture, Japan. Of these, 121 serum samples and 42 urine samples were collected from free-roaming, stray cats captured/accommodated at the Okinawa Prefectural Animal Protection and Control Center from June 2012 to November 2018, based on the Act on Welfare and Management of Animals. Cats were euthanized by carbon dioxide gas inhalation under the Act, not for this study, and all methods were performed in accordance with the American Veterinary Medical Association guidelines. Autopsy findings, body weight, sex, and capture area were recorded. Blood was collected in a serum separation tube by cardiocentesis and centrifuged at 1710 × *g* for 15 min to separate the serum. Urine was aseptically collected directly from the bladder using a syringe. Blood and urine collection from euthanized cats was conducted with permission from the Okinawa Prefectural Animal Protection and Control Center.

The other 120 serum samples were derived from the residual blood collected from free-roaming, stray cats during the free-roaming neutering program in the northern part of Okinawa Island from 2016 to 2018 carried out by a nonprofit organization. Serum was separated as described above, and the body weight, sex, and capture area of cats were recorded.

The study was carried out in compliance with the ARRIVE guidelines (https://arriveguidelines.org/).

### Antibody detection from cats

To detect anti-*Leptospira* antibodies in serum samples, MAT was performed using 13 reference strains of serogroups: Australis (serovar Australis), Autumnalis (Autumnalis and Rachmati), Ballum (Castellonis), Bataviae (Bataviae), Canicola (Canicola), Grippotyphosa (Grippotyphosa), Hebdomadis (Hebdomadis), Icterohaemorrhagiae (Icterohaemorrhagiae), Javanica (Javanica), Pomona (Pomona), Pyrogenes (Pyrogenes), and Sejroe (Hardjo). These reference strains were cultivated in Ellinghausen-McCullough-Johnson-Harris medium at 30°C^7^. Twenty-five microliters of twofold serially diluted serum samples [1:80–1:5120 by phosphate-buffered saline] were incubated with the same volume of leptospiral cultures for 3 h at 30 °C. The endpoint was determined by ≥ 50% decrease of free, unagglutinated leptospires compared with the control suspension^3^. Reciprocal MAT titer 160 was used for the cutoff antibody titer.

### DNA detection from cat urine samples

DNA was extracted from 200 µL urine using the QIAamp DNA Blood Mini Kit (Qiagen, Hilden, Germany) and subjected to nested PCR targeting *flaB* for the pathogenic *Leptospira* spp. First, 5 µl of extracted DNA were used for the first PCR using the primer set L-*flaB*-F1 5′-CTCACCGTTCTCTAAAGTTCAAC-3′ and L-*flaB*-R1 5′-TGAATTCGGTTTCATATTTGCC-3′ in a 50 µl reaction volume. Then, 1 µl of the first PCR product was added to 19 µl of the second PCR mixture with the primer set L-*flaB*-F2 5′-TGTGGACAAGACGATGAAAGC-3′ and L-*flaB*-R2 5′-AACATTGCCGTACCACTCTG-3′. The positive first PCR samples (FU18017, FU18027, and FU18028) were subjected to DNA sequencing using the BigDye Terminator v3.1 Cycle Sequencing Kit (Applied Biosystems, Foster City, CA, USA).

### MLST

MLST was performed for the *flaB*-positive DNA samples (FU18017, FU18027, and FU18028) and DNA samples extracted from nine strains of *Leptospira borgpetersenii* serogroup Javanica isolated from cats, a mongoose, black rats, and humans using the QIAamp DNA Blood Mini Kit, which were stored at − 80 °C at the Okinawa Prefectural Institute of Health and Environment^27,28^ (Table 2). MLST using seven housekeeping genes (*glmU*, *pntA*, *sucA*, *tpiA*, *pfkB*, *mreA*, and *caiB*) for the isolates was performed as previously described^40^. MLST for the *flaB*-positive DNAs, FU18017 and FU18028, was performed via nested PCR as previously described^42^. For FU18027, since five of the seven genes were not amplified using the original primer sets^41,42^, new primer sets were designed based on *L. borgpetersenii* sequences (accession numbers CP000350, CP012029, CP015044, CP015046, CP015048, CP015050, CP015052, CP015814, CP021412, CP026671, and CP033440) as described in Table 3. *glmU*, *sucA*, *tpiA*, *pfkB*, and *caiB* were amplified by nested PCR while *pntA* and *mreA* were amplified by the first PCR alone. Nucleotide sequences of the amplicons were determined using the BigDye Terminator v3.1 Cycle Sequencing Kit. The concatenated sequences were aligned in MEGA10, and STs were assigned through the MLST database (https://pubmlst.org/organisms/leptospira-spp). A minimum spanning tree (MST) based on the allelic profiles determined in this study and those of 182 *L. borgpetersenii* strains registered in the MLST database was created using BioNumerics Software (version 7.6; Applied-Maths, Sint Maartens-Latem, Belgium) with default settings (MST for categorical data).

**Table 3:** MLST primer sets for *Leptospira borgpetersenii* clone FU18027.

Locus	PCR	Primer name	Sequence (5′–3′)	References
glmU	1st	glmU-F_M_	AGGATAAGGTCGCTGTGGTA	^41^
	1st	glmU-R_M_	AGTTTTTTTCCGGAGTTTCT	^41^
	2nd	1-glmU-2F_M13	TGTAAAACGACGGCCAGTCGYATGAAAACGGATCAG	^42^
	2nd	1-glmU-2R_M13	CAGGAAACAGCTATGACCGGAAGRTARTATTCDCCCTG	^42^
pntA	1st	pntA-F-borg	GCCGGAGCAAATCTTGTATC	This study
	1st	pntA-R-borg	TGACCGATTACCGTTACCCC	This study
sucA	1st	sucA-F_M_	TCATTCCACTTYTAGATACGAT	^41^
	1st	sucA-R_M_	TCTTTTTTGAATTTTTGACG	^41^
	2nd	3-sucA-2F_M13	TGTAAAACGACGGCCAGTGCSGGTRATCATCWBATGG	^42^
	2nd	3-sucA-2R_M13	CAGGAAACAGCTATGACCGRAAWCCYTTYGCAAGATC	^42^
tpiA	1st	tpiA-F-borg	AAATCGCATGCGCAAAACGG	This study
	1st	tpiA-R-borg	GAGCGCTTGTATGTTATCCG	This study
	2nd	tpiA-F2-borg	CGCCGGAAACTGGAAAATGA	This study
	2nd	tpiA-R2-borg	TTCCGCAATTTCTTTCGCGC	This study
pfkB	1st	pfkB-F-borg	CCGGGAAGGTTTCCAAAGAC	This study
	1st	pfkB-R-borg	TAAAACCGTGGGTCAGTCCG	This study
	2nd	pfkB-F2-borg	GGAAAACGCCGGAATCCTTT	This study
	2nd	pfkB-R_M_	AGAACACCCGCCGCAAAACAAT	^41^
mreA	1st	mreA-F-borg	GGTGGAAAGATATGGCTCGC	This study
	1st	mreA-R-borg	TTCCTATCGCGGTCATGGAC	This study
caiB	1st	caiB-F	CAACTTGCGGAYATAGGAGGAG	^41^
	1st	caiB-R-borg	TCCGAGAGATCGGTAAATCG	This study
	2nd	7-caiB-2F_M13	TGTAAAACGACGGCCAGTCTTKCTTCRATYTTGGCG	^42^
	2nd	7-caiB-2R_M13	CAGGAAACAGCTATGACCAMCGATATGTWAYMGGRGTT	^42^
glmU	1st	glmU-F_M_	AGGATAAGGTCGCTGTGGTA	^41^
	1st	glmU-R_M_	AGTTTTTTTCCGGAGTTTCT	^41^
	2nd	1-glmU-2F_M13	TGTAAAACGACGGCCAGTCGYATGAAAACGGATCAG	^42^
	2nd	1-glmU-2R_M13	CAGGAAACAGCTATGACCGGAAGRTARTATTCDCCCTG	^42^

MLST, multilocus sequence typing.

### WGS

Genomic DNA from the six strains in Table 2 was prepared as described above. Genomic DNA libraries were prepared using the Nextera XT DNA Library Prep Kit (Illumina, San Diego, CA, USA) according to the manufacturer’s instructions and sequenced on MiSeq (Illumina) with 300 bp paired-end reads. Core genome single nucleotide variants (SNVs) were extracted using BactSNP v.1.1.037^43^ with the genome of *L. borgpetersenii* serogroup Javanica serovar Ceylonica strain Piyasena as the reference (GenBank accession no. CP026671.1 and CP026672.1). For phylogenetic analysis, SNVs in the recombinogenic regions detected using Gubbins version 2.3.4^44^ and those in the repetitive regions of the Piyasena genome identified using MUMmer v.3.2259^45^ were excluded. Phylogenetic relationships were determined by reconstructing a phylogenetic tree via the maximum likelihood method using IQ-TREE^46^ with 1000 ultrafast bootstrap replicates. The data have been deposited with links to BioProject accession number PRJDB10861 in the DDBJ BioProject database.

### Statistical methods

To define the capture areas, the island was divided into northern, central, and southern areas. Cats were categorized according to three age groups based on weight as previously described ^47^: male: kitten, < 1.0 kg; juvenile, 1–2.4 kg; and adult, ≥ 2.5 kg and female: kitten, < 1.0 kg; juvenile, 1–1.9 kg; and adult, ≥ 2.0 kg. Associations of seropositivity with capture area, sex, and age based on weight were analyzed using χ^2^ test or 2 × 3 Fisher’s exact test and χ^2^ test for trend.

### Ethics declarations

No ethical approval was required as the samples were collected from cats sacrificed under an act or derived from residual blood from health examinations.

## Supplementary Information

Supplementary Information.

## Data Availability

The *flaB* sequences have been deposited in a public database (DDBJ accession numbers LC596932–LC596934). The WGS data have been deposited with links to BioProject accession number PRJDB10861 in the DDBJ BioProject database.
