# Supplementary material for: Molecular and serological epidemiology of Leptospira infection in cats in Okinawa Island, Japan
Source: Sci Rep. 2021 May 14;11:10365. doi: 10.1038/s41598-021-89872-3 (PMC8121857; doi:10.1038/s41598-021-89872-3)
Supplement: Supplementary file 1 — Supplementary Information. [file 41598_2021_89872_MOESM1_ESM.pdf]

Molecular and serological epidemiology of *Leptospira* infection in cats in Okinawa Island, Japan

Tetsuya Kakita, Yumani Kuba, Hisako Kyan, Sho Okano, Masatomo Morita, Nobuo Koizumi

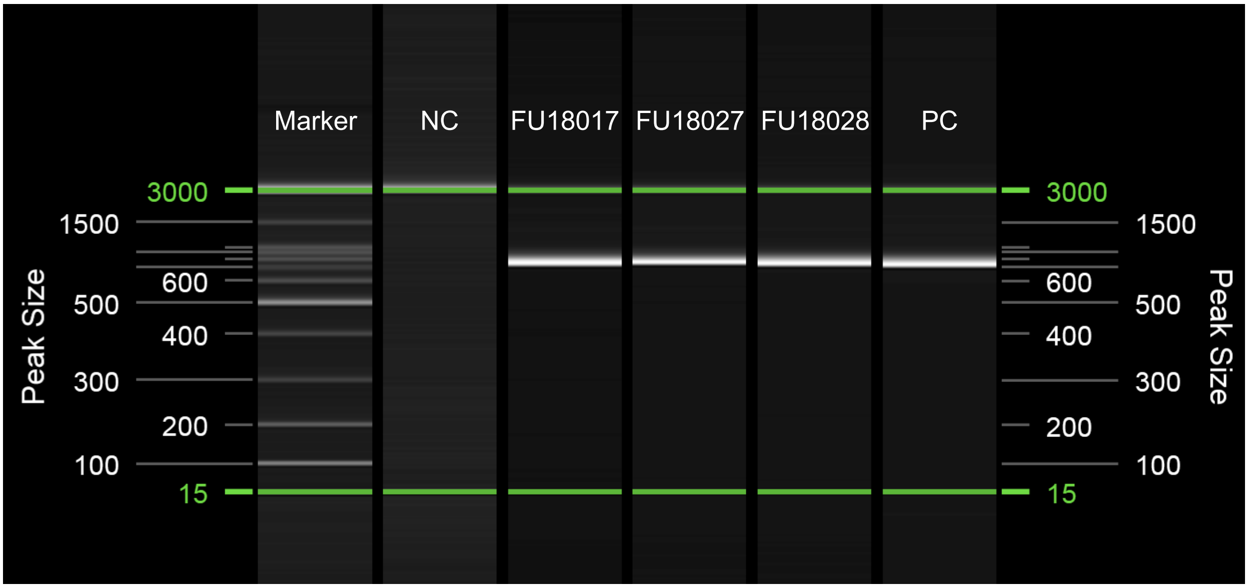

Supplementary Figure S1. Capillary electrophoresis of *flaB*-nested PCR amplicons.
NC; negative control (no template DNA), PC; positive control (*L. interrogans* serovar Hardjo strain Hardjoprajitno genomic DNA)
